# Editorial: Current Challenges in Immune and Other Acquired Cytopenias of Childhood

**DOI:** 10.3389/fped.2016.00003

**Published:** 2016-02-02

**Authors:** Sujal Ghosh, Markus G. Seidel

**Affiliations:** ^1^Department of Pediatric Oncology, Hematology and Clinical Immunology, Center of Child and Adolescent Health, Medical Faculty, Heinrich-Heine University, Düsseldorf, Germany; ^2^Molecular and Cellular Immunology Section, University College London – Institute of Child Health, London, UK; ^3^Research Unit for Pediatric Hematology and Immunology, Division of Pediatric Hematology-Oncology, Department of Pediatrics and Adolescent Medicine, Medical University Graz, Graz, Austria

**Keywords:** immune thrombocytopenia (ITP), Evans syndrome, pancytopenia, autoimmune disease, immune dysregulation, primary immunodeficiency, thrombopoietin receptor agonists, autoimmune hemolytic anemia

**The Editorial on the Research Topic**

**Current Challenges in Immune and Other Acquired Cytopenias of Childhood**

Chronic immune thrombocytopenia (ITP), Evans syndrome (ES), aplastic anemia, etc., are descriptive terms for immune-mediated cytopenias in pediatric hematology that may be summarized as a group of disorders of immune dysregulation based on ill-defined poly- and epigenetic diatheses toward autoimmunity and monogenic primary immunodeficiencies (PIDs); a growing number of which is being identified with next-generation sequencing technologies. The historical view, that cytopenia in the context of these diseases is “acquired,” “idiopathic,” or termed “primary” because cytopenia may be the first and only manifestation at early age, is challenged by the emerging recognition of underlying pathomechanisms and predispositions. Similarly, patients with congenital bone marrow failure syndromes may present without previous syndromic features later during childhood or adolescence and contribute to the wide spectrum of differential diagnosis of cytopenia in childhood. In addition to the diagnostic complexity and prognostic uncertainty, the therapeutic approach to patients with these conditions remains undetermined in many situations. In severe cases, allogeneic hematopoietic stem cell transplantation may be the option of choice; other conditions might require temporary immunosuppression or even no treatment at all. Although the indication for stem cell transplantation will always predominantly depend on the clinical course and status of a patient, the definition of a clear-cut pathomechanism potentially offers a guidance toward targeted therapy approaches.

Autoimmunity and cytopenias may occur in the context of many types of PID, whether belonging to the combined immunodeficiencies, to those with predominant antibody deficiency, to syndromic PIDs (e.g., Wiskott–Aldrich or 22q11 deletion syndrome), to the category of PIDs with immune dysregulation [e.g., autoimmune lymphoproliferative syndrome (ALPS), immune dysregulation, polyendocrinopathy, enteropathy, X-linked (IPEX) syndrome], as well as to humoral and cellular defects of the innate immune system (1, 2). In addition to classical PIDs highly associated with autoimmunity and autoimmune cytopenias such as ALPS or common variable immunodeficiency (CVID), an increasing number of PID syndromes have been described recently to be associated with immune dysregulation and symptoms of autoimmunity such as immune cytopenia. The phenotype in 9 of 13 newly discovered monogenic PIDs published within the last year include cytopenia, immune dysregulation, autoimmunity, and/or autoinflammation as their main manifestation (1, 3, 4), adding to at least 8 of 19 novel PIDs the year before [(5); Al-Herz et al.]. Yet, this fact has not universally been translated into the diagnostic work-up of cytopenia in childhood.

The present research topic on immune and other acquired cytopenias of childhood at *Frontiers in Pediatric Hematology and Immunology* encompasses reviews, original studies, and single-case observations as well as management strategy papers that address the current challenge how to deal with these disorders. Erlacher and Strahm review in depth aspects and differential diagnosis of pancytopenia in childhood, ranging from inherited bone marrow failure syndromes to extrinsically acquired (e.g., infection-associated) defects of hematopoiesis; from autoimmunity to hemophagocytic syndromes and malignancies (e.g., acute lymphoblastic leukemia and myelodysplastic syndromes).

Classical inherited bone marrow failure syndromes, e.g., Fanconi anemia, Dyskeratosis congenita, Shwachman Bodian Diamond Syndrome, and Diamond Blackfan anemia, are based on defects of DNA and telomere maintenance or ribosome function. However, immune dysregulation and autoimmunity often have a rather unclear and heterogenous etiology. Polygenetic defects or polymorphisms as well as an array of environmental factors are known to contribute to a predisposition to autoimmunity. In susceptible individuals, autoantibodies may be produced secondarily to infections or other exogenous triggers due to cross-reactivity (molecular mimicry). Pathologic processing of cell debris can lead to presentation of self-antigens to the immune system like anti-glycoprotein IIa/IIIb antibodies in ITP or anti-double strand DNA antibodies in systemic lupus erythematosus (SLE). Among several PID disorders that lead to a predisposition toward autoimmunity, ALPS, most often due to defective Fas-mediated lymphocyte apoptosis and impaired T cell maturation, is a classical PID leading to autoimmune cytopenia, splenomegaly, and lymphoproliferation, with splenic sequestration sometimes contributing to cytopenia. Furthermore, the heterogeneous group of CVID is highly associated with autoimmune cytopenia due to autoantibody formation based on defective B cell selection and maturation. CVID and ALPS may be ruled out on the basis of relatively routine basic immunological tests. The large group of combined immunodeficiencies is generally associated with a lack of naive T cells and an oligoclonal T cell repertoire, which predisposes these patients to autoimmunity in addition to infections. Wiskott–Aldrich and 22q11 deletion syndromes are linked to defective regulatory T (Treg) cells and impaired T cell development, and may be excluded by detection of additional clinical syndromic features, other routine laboratory parameters, impaired *in vitro* lymphocyte proliferation, and molecular genetic tests; whereas patients with IPEX-(like) syndromes have a primary Treg defect and most often present with enteropathy, multi-organ autoimmunity, and show reduced or absent Treg cell function and diminished STAT5 phosphorylation. Recently, homozygous loss-of-function mutations in the LRBA gene (3, 6, 7) as well as haploinsufficiency of CTLA-4 (8, 9) gain-of-function of PI-3-kinase (10, 11) or of STAT3 (12, 13) showed in part ALPS-like phenotypes with autoimmune cytopenias. In line with these observations, an increasing number of patients with autoimmunity including cytopenias will be referred to genetic analysis to find new causative genes. Rao highlights the experience of the NIH with one of the largest ALPS cohorts in the world, emphasizing the need of effective immune suppression. One of the main lessons from the past decades taught us to avoid splenectomy. Furthermore, Aladjidi et al. report the results from French OBS’CEREVANCE, an observational cohort gathering data on children with ES, chronic ITP and autoimmune hemolytic anemia (AIHA). One hundred fifty-six patients with ES were analyzed; interestingly, in 13 patients SLE was diagnosed, but ALPS was diagnosed only in 3 patients. Thirty percent of all patients were classified as “primary” forms because cytopenia remained the only symptom; in 60% of the patients, the authors observed additional clinical or biochemical features to term this fraction as “unclassified” (Aladjidi et al.). One major red flag for pediatricians: 10% of all patients died at a median age of 14.3 years either due to hemorrhage or infections with the unknown participating role of immunosuppressive treatment. Thus, a “wait-and-see” strategy in ES seems not to be justified for a long period.

Phenotypic variations of diseases linked to (pan-)cytopenia are shown in the case report of Karastaneva et al. Two unrelated patients with Fanconi anemia developed rather untypical ITP, but showing a normal marrow. Management of ITP was accomplished with intravenous immunoglobulins (IVIG) and danazol. This rather simple and non-toxic ITP treatment warrants evaluation of autoimmune phenomena in other bone marrow failure syndromes.

Although in most patients with ITP first-line treatment usually leads to remission, the application of thrombopoietin agonist is warranted in patients with refractory and chronic forms of ITP. Garzon and Mitchell review the use of Eltrombopag and Romiplostim, which depicts a change in treatment paradigm, as an immunosuppressive regime is avoided, and these drugs are well tolerated. Finally, Koster et al. report the experience of a single hematooncological unit with two cases of a rare infectious disease, namely Leishmaniosis that led to severe cytopenia. The reported incidence of Leishmaniosis among pediatric patients with cytopenia is extremely low, having said that the experience of the reporting hematooncologial unit in central Germany with two cases in 10 years shows how coincidental rare events might happen. Hemophagocytosis (detected after repeated marrow puncture) and polyclonal B cell activation (false positive antibodies for other infectious diseases) are hallmarks of Leishmaniosis.

This research topic has collected a range of contributions focusing on diagnostic and therapeutic challenges, reaching from scientific progress in the immunological and hematological context of cytopenias to clinical challenges such as the experience with novel treatment approaches and lessons from patient registries. Despite tremendous progress has been made with next-generation sequencing techniques applied in the diagnostic work-up and the availability of novel potent immunosuppressive drugs and hematopoiesis stimulating agents for the treatment recently, clinical challenges remain for those dealing with the so-called “acquired” cytopenias of childhood.

## Author Contributions

Both authors wrote the manuscript.

## Conflict of Interest Statement

The authors declare that the research was conducted in the absence of any commercial or financial relationships that could be construed as a potential conflict of interest.
